# Dental Prophylaxis

**Published:** 1908-09-26

**Authors:** A. Louis George


### Dental Department.

### DENTAL PROPHYLAXIS.

By A. LOUIS GEORGE, L.D.S.Eng.

In a previous article we endeavoured to show that dental caries is invariably brought about by the action of bacteria; that our high state of civilisation and artificial life, as compared with those of primitive man, tend to assist bacteria in their work of disintegration and decay of tooth tissue. The question now to be considered is, Can anything be done to prevent dental caries; is there any reasonable line of action we can lay down for the guidance of the individual, the conscientious following of which will absolutely prevent this trouble? The answer is No.

Let us take a hypothetical case. A patient of average intelligence and means brings a child twelve years old to us, who exhibits signs of dental caries in the four first permanent molars. The first question we are asked is, Why have the teeth decayed? The next question should be, What can you do to prevent other teeth from decaying? The answer to the second question comprises the whole of dental prophylaxis.

The cause of dental caries being microbic, it follows that if we could prevent the access of bacteria to the teeth we should prevent caries. But bacteria are so universally present that their exclusion from the mouth is without the bounds of possibility. We have shown that bacteria find a lodgment in pits, grooves, and flaws in the enamel surface and between adjacent surfaces and round the points of impingement of one tooth against another. Now though we cannot prevent the access of bacteria to the mouth, we can to some extent prevent their lodgment in these suitable places. To do this we want a fairly stiff tooth-brush and a good powder, and we want method in their application. It is very little good to insert a tooth-brush dipped in powder between the buccal and labial surfaces of the teeth and the cheek and lips, and with the eyes shut make half a dozen quick passes across the teeth on each side of the mouth, with a little extra vigour reserved for the front ones because they show. What is wanted is a systematic cleansing as far as possible of each individual tooth, with very special precautions to reach those places difficult of access. This is rendered easier by using, in addition to the ordinary tooth-brush, in which the bristles are at right angles to the handle and extend for an inch or an inch and a half along the shaft, a brush made to form a little somewhat cone-shaped tuft at the extreme end of the shaft, the bristles still at right angles to the handle. This brush gets into places where the former cannot reach. In addition to this thorough brushing, dental silk should be used. Where possible, it should be gently forced between the teeth, and the sides of each tooth cleaned. Sometimes it is not possible to force the silk between the teeth because they are in such close apposition; in this case the silk can often be threaded through the space between the teeth near the gum margin; it can then be drawn through and through, reaching as far up as possible. Further, in addition to the tooth-brush, powder, and dental silk, a mild alkaline antiseptic mouth-wash should be used.

A good tooth-powder should be gritty, but almost imperceptibly so: it should be just gritty enough to remove the film of greasy material which clings round the teeth and to dislodge the bacteria which have established a hold. It should be alkaline to neutralise the acid formed by the fermentative processes of the micro-organisms; it should be antiseptic to render the environment of the bacteria as adverse to their development as possible; it should be pleasant to the taste and agreeable to the eye and not too costly. A tooth-powder which fulfils all these requirements, which we have found in every way satisfactory, and which patients like well is:—

| | |
|---|---|
| Saponis hisp. ... ... ... ... | 2 drs. |
| Pulv. Iridis. ... ... ... ... | 3 drs. |
| Os. Sepiæ pulv. ... ... ... ... | 2 drs. |
| Magnes. carb. pond. ... ... ... | 2 oz. |
| Cretæ præcip. ... ... ... ... | 2 oz. |
| Otto Rosæ ... ... ... ... | 8 mns. |
| Ol. Eucalypt. ... ... ... ... | 8 mns. |

Chemists will put this powder up in shilling boxes. With regard to mouth-washes, the essentials are that the mouth-wash shall contain an effective antiseptic, and that the cost shall be low; for if the wash is to be used by every member of a moderate-sized family every day or twice a day all the year round, and if the cost is not decidedly small, before long it will be discontinued on the score of expense. The following is a good efficient mouth-wash, but its expense is a bar to its general use:—

| | |
|---|---|
| Thymol ... ... ... ... ... | 2 gr. |
| Acidi benzoic. ... ... ... ... | 45 gr. |
| Tinct. Eucalypt. ... ... ... ... | ½ oz. |
| Olei Gaultheriæ ... ... ... ... | 25 gr. |
| Spirit. Rectificat. ... ... ... ... | 4 dr. |

Potassium permanganate is cheap, convenient to keep, and readily soluble. If about three crystals are placed in a tumbler of water, the mouth-wash is ready to use. It is a disinfectant, deodorant, and slightly antiseptic, but it is not very pleasant to taste. Tinct. of myrrh is a good wash for the mouth; it is antiseptic, astringent, does not mix with water, but can be diffused through it, is a little pleasanter than potassium permanganate, but not so cheap.

A word must be said as to the times for cleaning the teeth. The best time is undoubtedly before going to bed at night. There is then plenty of time, and the operation need not be perfunctory and hurried; moreover, during sleep the tongue and muscles of the cheek are still, and the flow of saliva is not maintained, food clings to the teeth, and micro-organisms are free to develop. While the main scouring is to be done at night, the teeth should be given another brushing *after* breakfast.

Having given our hypothetical parent these instructions as to dental prophylaxis, we should tell him that though he may carry out our instructions conscientiously and to the letter, still his child's teeth may decay; and we should urge to the utmost another precaution he should take, and one quite as important, if not more so, than all the others—that is, periodic inspection by the dentist. For we should point out that while by no known means can we absolutely prevent decay, we can certainly prevent the child from losing its teeth. A tooth does not decay *en masse*; all decay starts at one or more small points, which increase in size as time goes on. If the tooth starts in two or more places at the same time, these get larger simultaneously, eventually join together, and the tooth collapses. Now by inspection three times a year at the hands of a dentist the holes can be discovered while they are small; they can then be easily filled without much pain and with permanent results, and we can assure this parent that a tooth scientifically filled with suitable material is in all respects as good as a tooth that has had nothing the matter with it; and we can promise him that if he does as we have advised, his child at the age of twenty-one will have every tooth in its head, will not have suffered from toothache, and will have in every respect a serviceable set of teeth.

